# Stakeholders’ perceptions of ways to support decisions about health insurance marketplace enrollment: a qualitative study

**DOI:** 10.1186/s12913-016-1890-8

**Published:** 2016-11-08

**Authors:** A. J. Housten, K. Furtado, K. A. Kaphingst, C. Kebodeaux, T. McBride, B. Cusanno, M. C. Politi

**Affiliations:** 1 Division of Public Health Sciences, Department of Surgery, Washington University in St. Louis School of Medicine, 660 S. Euclid Ave, Campus Box 8100, St. Louis, MO 63110 USA; 2 George Warren Brown School of Social Work, Washington University in St. Louis, Campus Box 1196, One Brookings Drive, St. Louis, MO 63130-4899 USA; 3 Department of Communication, Huntsman Cancer Institute, University of Utah, 255 S Central Campus Dr., Room 2400, Salt Lake City, UT 84112 USA

**Keywords:** Affordable care act, Health insurance, Decision making, Decision support, Qualitative research

## Abstract

**Background:**

Approximately 29 million individuals are expected to enroll in health insurance using the Patient Protection and Affordable Care Act (ACA) Marketplace by 2022. Those seeking health insurance struggle to understand insurance options and choose a plan that best suits their needs.

**Methods:**

We interviewed stakeholders to identify the challenges associated with the ACA Marketplace health insurance enrollment and elicited feedback about what to include in health insurance decision support tools. Interviews were transcribed and themes were identified using inductive thematic analysis.

**Results:**

Stakeholders stated that consumers felt frustrated by unclear terminology, high plan costs, and complex calculations required to assess costs. Consumers felt anxious about making the wrong choice and being unable to change plans within a calendar year. Stakeholders recommended using plain language tables defining complex terms, grouping information, and using engaging graphics to communicate information about health insurance. Stakeholders thought that narratives of how others made decisions about insurance might be helpful to consumers, but recommended that they be tailored to the needs of specific consumers.

**Conclusion:**

Strategies that clarify health insurance terms using plain language and graphics, acknowledge concern associated with making the wrong choice, calculate and enable cost comparison, and tailor information to consumers’ unique needs could benefit those enrolling in ACA Marketplace plans, Narratives developed should be simple and inclusive enough for diverse populations.

## Background

Approximately 20 million people have gained access to health insurance through the Patient Protection and Affordable Care Act (ACA) as of early 2016 [1]. Since the ACA’s implementation, the rate of uninsured individuals in the United States has dropped by 43 % (20.3 to 11.2 %) [1, 2]. Estimates are that 29 million individuals will enroll in a health insurance plan using the ACA Marketplace by 2022 [3]. While all plans in the ACA Marketplace meet minimum coverage standards [4], plan features such as premiums, deductibles, copayments, and provider and hospital networks vary. Understanding these plan features and selecting a plan that meets health and financial needs can be challenging. Those with limited health insurance literacy and numeracy may find this process especially daunting [5–8]. The growing number of people with limited health literacy and numeracy enrolling in health insurance demonstrates a need to address the challenges associated with insurance enrollment [5].

Limited understanding of terms and concepts related to health insurance may lead to suboptimal plan choices [8]. Those who select health insurance plans that do not fit their needs may not seek needed care because of concerns over cost and coverage [9]. Despite recommendations for tools that help consumers select optimal health insurance plans, few consumer-friendly tools have been incorporated into Marketplace enrollment [10–18]. Presenting information using clear, personalized communication can facilitate decision making by emphasizing the meaning, relevance and importance of the information to consumers [8, 10, 11, 14, 15, 19, 20]. Further, use of clear communication strategies may help individuals select health information that best meets their needs [8, 19].

The goal of the current study was to conduct semi-structured interviews with key stakeholders to better understand the challenges associated with enrolling in health insurance using the ACA Marketplace. We also explored and how our previously developed health insurance tool [4, 8] could facilitate health insurance decision and/or how it could be improved to address challenges associated with Marketplace enrollment.

## Methods

### Study design

We conducted semi-structured qualitative interviews with key stakeholders, including certified application counselors (CACs), health care providers, Marketplace consumers, and health policy experts. We asked stakeholders about their perceptions of the challenges associated with health insurance enrollment in the ACA Marketplace and information essential to include in health insurance decision support tools. Next, we showed our stakeholders strategies previously developed to support health insurance decisions [4, 8]: the first strategy presented plan-specific information on a plain language table, the second was a visual strategy using graphics and breaking information into smaller pieces and the third was a narrative strategy with both the plain language table plus short vignettes about how people might approach health insurance decisions. The study was approved by the Washington University Human Subjects Research Protection Office.

### Participants and recruitment

We conducted interviews with participants from all four stakeholder groups described above. These groups were selected because of their insight into various aspects of the population eligible for the Marketplace and their decision-making process. Stakeholders were recruited through purposive and snowball sampling, over-recruiting CACs because of their familiarity with challenges and opportunities to improve ACA enrollment. Policy experts, CACs, health care providers and Marketplace consumers were identified through their affiliation with key organizations and partners from previous work on this topic [4, 8]. These organizations included area health clinics, community centers, and social service organizations. Initial contacts at these organizations were encouraged to forward the invitation to participate in the study to their colleagues. Consumers were also recruited through online advertisements.

### Theoretical framework

We based our interview guide on a model of implementation research [20] suggesting ways to identifying factors that affect broad implementation. The model suggests that researchers first engage stakeholders in identifying evidence-based intervention strategies; next engage them to revise those strategies and based on perceptions of multiple levels of outcomes; then test those strategies across a range of outcomes including at the individual and policy level. In this investigation, we engaged stakeholders to revise previously-developed strategies and elicited feedback to modify an evidence-based intervention. This multi-level strategy differs from linear approaches which traditionally investigate demonstration and implementation as the final stage of scientific inquiry [21].

### Interviews

At the beginning of the semi-structured interviews, participants provided information about their role at their institution (for all but Marketplace consumers), how they approach health insurance decisions, how they think the health insurance decision-making process could be improved, and the amount and kind of information consumers seek about health insurance. Next, participants were shown our health insurance decision tools and were encouraged to give open feedback about those tools (i.e., likes, dislikes, suggestions for modifications). CACs, policy experts, and health care providers were asked to speak from both the perspective of their uninsured clients as well as from their own perspective. Participants were compensated $20 for their time.

Interviews were conducted by two members of the research team (KF and CK) trained in qualitative interviewing methods. Interviews were audio recorded and field notes were taken. Data were then transcribed, de-identified, and reviewed for analysis.

### Data analysis

Interviews were coded using QSR NVivo 10 and analyzed with an inductive thematic analysis approach. Three members of the research team (CK, KF, and MP) first reviewed the transcripts and developed a preliminary codebook based on emerging results and the interview guide. Team members (CK and KF) then coded the first five transcripts, discussed the coding process, and further revised and refined the codebook with the principal investigator (MP). Coders discussed inconsistent codes and if consensus could not be reached, a third coder (MP) helped resolve discrepancies. Once the two coders reached consensus (kappa >0.70), they coded the remaining transcripts independently using the revised codebook. Themes were identified based on both the frequency of codes and emotive force conveyed when discussing the material as determined by the reviewers.

## Results

Forty interviews were conducted at which point we reached thematic saturation. The final study sample consisted of 4 policy experts, 29 CACs, 2 health care providers, and 9 consumers (some participants self-identified as multiple stakeholder types). Table 1 shows the demographic characteristics of participants. Interviews took place in January and February 2015. All interviews lasted between 30 and 60 min. The main themes are described below with illustrative quotes and in Table 2. Participant (P) number and type are noted with their associated comments.

**Table 1:** Participant Demographic Characteristics

Participant Demographics (*N* = 40)	
Characteristic	*n* (%)
Gender	
Male	14 (35.0)
Female	26 (65.0)
Stakeholder Type^a^	
Uninsured/Recently Uninsured	9 (22.5)
CAC	29 (72.5)
Provider	2 (5.0)
Policy Expert	4 (10.0)
Time of Employment (*N* = 31)^b^	
< 1 year	11 (35.5)
1–2 years	12 (37.5)
3–5 years	4 (12.9)
6–10 years	3 (9.7)
> 10 years	1 (3.2)
Highest Level of Education	
HS Diploma or equivalent or less	0
Technical training or certification	1 (2.5)
Some college	4 (10.0)
College degree	22 (55.0)
Graduate/Professional degree	13 (32.5)
Latino or Hispanic	
Yes	2 (5.0)
No	38 (95.0)
Race	
Caucasian	27 (67.5)
African American	7 (17.5)
Asian	2 (5.0)
Native American or Alaskan Native	0
Native Hawaiian or other Pacific Islander	0
Other	3 (7.5)
Missing	1 (2.5)

^a^Participants were allowed to select more than one stakeholder type. One participant reported being both a CAC and uninsured/recently uninsured, two participants reported being a CAC and a healthcare provider, and one CAC reported being a policy expert

^b^This question was only asked of institutional stakeholders

**Table 2:** Themes, subthemes and example quotes for the qualitative analysis

Themes	Subthemes	Example quotes
Perceptions of Challenges with Enrollment	Fears about Making a Suboptimal Insurance Choice	“Um they have trouble making health insurance decisions because sometimes they have a time limit, time limit to get health insurance so it’s like they feel like they’re being rushed and then, once they get in a health plan, it’s like, ‘Can I change up the plans or do I have to stay in it for a certain amount of time or…?’ Cause some of my clients ask me, ‘Well, what if I pick this health plan and in 60 days from now I don’t like it?’” (P3, CAC)
		“So, and I have had people come back that, there’s one plan in particular, that they took that plan last year and they went somewhere out-of-network and got all these bills and they got upset about it so they changed this year.” (P6, CAC)
		“Because I was so worried about my chronic care I didn’t worry if something might pop up [that’s new]…always afraid of making the wrong choice, I guess. Cause once I make it, it’s done, I imagine…until next year.” (P34, Consumer)
	Lack of Knowledge and Financial Literacy among Consumers	“It’s complicated…There are a lot of choices and then there are terms that are unfamiliar or they mean something different in health care than they do in other industries. So, like…the deductible. So a lot of people think, most people are familiar with car insurance. So they think deductible and they think every time you have an accident you have to pay out your deductible and it’s different for health insurance…people get really confused about that term.” (P37, Policy)
		“The summary of benefits is not at all clear for most consumers… I don’t think any consumer can figure out on their own, even with the highest level of education…” (P18, CAC)
		“Uh, the only thing I see clients struggle with is, they, they tend to think about a lot… how to make those payments.” (P12, CAC)
		“Um, people need to have some better information… [about] how to make financial decisions…because this stuff is pretty complicated. So helping them think about, um, so you know what a premium is… [and] how does the premium affect the co-pay and your lifetime use and all those things…and so we need to explain that part a whole bunch better…” (P3, CAC)
		“I guess in general, just kind of like…the pros and cons, um, to help people understand that like, no plan’s perfect. And, um, there’s a choice to make and there’s a tradeoff.” (P1, Policy)
What to Include in Decision Support Tools	Importance of Health Insurance Premium	“Um, but in, but in most cases, I’d be looking for, um, what to expect, what am I expected to pay month to month.” (P2, CAC)
		“For 80 % of consumers or more I’d the premium they’d say, “the premium rules,” and that rules the day…” (P18, CAC)
		“I think like anybody one of the things you have to think about is what’s affordable, what can I afford monthly premium wise, probably even more so than the…deductible.” (P38, Consumer)
		“[Client’s priorities should be] … all the pricing of course which would be not only premiums but also deductible and the max out of pocket which not all consumers are immediately aware of.” (P29, CAC)
		“You know um a lot of people don’t even know that it’s, their deductibles and so, you know, insurance uh premiums may be low but the deductible might be high.” (P9, CAC)
	Importance of Network Coverage Differs Among Consumers	“Um, or they’re just like, “oh I haven’t been [to the doctor] in a lot of years,” I don’t care if I have to change. Like most people are pretty indifferent about that [network list], unless they, like, actually have…health issues that they’re concerned about.” (P6, CAC)
		“Uh, I would be very upset if I couldn’t keep my doctor. He’s a great guy. Sometimes when we don’t have money he just brings us in and sees us anyway.” (P32, Consumer)
		“Yeah, wherever you want to be able to go, um are there facilities or physicians in your area, which um here in St. Louis it’s not too much of an issue but it can be a challenge again with people with complicated health concerns that those will fit into the network and the plan.” (P19, CAC)
Ways to Modify Strategies	Addressing Challenges through Plain Language Principles	“Ok, I think the language needs to be simplified. It’s very, very complicated to most people…even me… I’m like “What are they talking about here?” (P5, Policy)
		“I think this is a really traditional model, that’s what you get when you go to, look in the insurance company and everybody disregards that because it’s just too complex and so breaking them down in a smaller format makes sense.” (P3, Policy)
		“…having it broken down into smaller charts would probably make it less intimidating for people to read.” (P6, CAC)
		“… And just visually… I think it’s just easier for people, [to] get one subject, um, at a time… I kind of, vote for that.” (P17, Provider)
		“Um, I would want to see the basics about each plan…otherwise it would just be way too overwhelming and hard to keep all of the information straight.” (P4, Policy)
		“Hmm, (laughs), I think more visual aids would be helpful. Um, so something that’s not just words on the screen…” (P4, Policy)
		“I like the picture, I like the white space. Um, very simple, small bit of information on the first page.” (P37, Policy)
	Including Comprehensive, Tailored Narratives to Support Insurance Choices	“I think that looking at these, um, the way that these vignettes walk you through somebody’s decision process could be very helpful.” (P33, Consumer)
		“Yeah I like, I like this [narrative], this is exactly what I was saying I’d like to see somebody, have an example, somebody that’s got a similar situation and find out what they did. Um, if that’s the one they chose well I can look at that and say “yes, that makes sense, you know, that’s what I probably ought to do as well.” (P34, Consumer)
		“… I think a person like that probably isn’t going to gravitate toward a big block of text and read it. Um…although I think the examples themselves are useful… I just don’t know if I see people actually reading them… especially when they already are taking in so information and they are pressed for time…” (P29, CAC)
		“A lot of people have complex enough family situations that there may or may not be just one of the vignettes that matches perfectly with theirs and so then they have to put them all side by side and make those, make those decisions and I think that’s hard.” (P3, Policy)
		“…[maybe] include someone who is disabled…include someone who is going to be graduating off their parents insurance at 26 or 27…include someone who, because of their citizenship status does not qualify to get Medicaid then they will be under their parents…maybe possibly even someone who has a kid in college in a different state but their permanent residence is with their parents, what kind of plan would you buy for that?” (P27, CAC)
		“[what about] same sex couples....and then the invincible young man kind of thing…and a multi-generational family, you know, where there’s’ like a, mom, or a grandma…we see so many of those types of families…there are those families out there.” (P10, CAC)
		“Now so one of my reactions is, my only, so if I had all of these put in front of me, I would actually want somebody to sort of hand me the one (laughs) that fits my situation closest. Um, and so I don’t know if that’s, once again, if you’re sitting with an assister or someone that’s helping you with this, instead of putting all five of these in front of me, if I know I little bit about you as the client, maybe I’m only putting one or two of these in front of you that best [fit] sort of your family situation.” (P37, Policy)

### Perceptions of challenges with enrollment

#### Fears about making a suboptimal insurance choice

Stakeholders talked about consumers’ fear of making the wrong choice and the potential negative consequences for being enrolled in that plan for an entire year.

*“Um they have trouble making health insurance decisions because sometimes they have a time limit, time limit to get health insurance so it’s like they feel like they’re being rushed and then, once they get in a health plan, it’s like, ‘Can I change up the plans or do I have to stay in it for a certain amount of time or…?’ Cause some of my clients ask me, ‘Well, what if I pick this health plan and in 60 days from now I don’t like it?’” (*P3, CAC)

*“So, and I have had people come back that, there’s one plan in particular, that they took that plan last year and they went somewhere out-of-network and got all these bills and they got upset about it so they changed this year.”* (P6, CAC)

Moreover, an uninsured participant stated anxiety over committing to one insurance plan for a 12-month period that may not have the desired features to manage a chronic condition:

*“Because I was so worried about my chronic care I didn’t worry if something might pop up [that’s new]…always afraid of making the wrong choice, I guess. Cause once I make it, it’s done, I imagine…until next year.”* (P34, Consumer)

#### Lack of knowledge and financial literacy among consumers

Stakeholders identified unfamiliar health insurance terminology as one of the biggest challenges with enrollment:

*“It’s complicated…There are a lot of choices and then there are terms that are unfamiliar or they mean something different in health care than they do in other industries. So, like…the deductible. So a lot of people think, most people are familiar with car insurance. So they think deductible and they think every time you have an accident you have to pay out your deductible and it’s different for health insurance…people get really confused about that term.”* (P37, Policy)

*“The summary of benefits is not at all clear for most consumers… I don’t think any consumer can figure out on their own, even with the highest level of education…as to which charges apply before deductible and which charges apply after the deductible…”* (P18, CAC)

Affordability was also identified as a major barrier to enrollment:

*“Uh, the only thing I see clients struggle with is, they, they tend to think about a lot… how to make those payments.”* (P12, CAC)

Financial literacy was highlighted during interviews as an enrollment challenge. Stakeholders supported the need to use plain language and clear explanations to facilitate cost and insurance plan feature comparisons. As one participant stated:

*“Um, people need to have some better information… [about] how to make financial decisions…because this stuff is pretty complicated. So helping them think about, um, so you know what a premium is… [and] how does the premium affect the co-pay and your lifetime use and all those things…and so we need to explain that part a whole bunch better…”* (P3, CAC)

Additionally, the need to make cost-related tradeoffs requiring multifaceted cost comparisons was identified as an important component of decision making. As a policy participant stated:

*“I guess in general, just kind of like…the pros and cons, um, to help people understand that like, no plan’s perfect. And, um, there’s a choice to make and there’s a tradeoff.”* (P1, Policy)

### What to include in decision support tools

#### Importance of health insurance premium

Stakeholders provided feedback on the most important features to include in decision support tools. Stakeholders indicated that the costs of premiums are often central to consumers’ decisions:

*“Um, but in, but in most cases, I’d be looking for, um, what to expect, what am I expected to pay month to month.”* (P2, CAC)

*“For 80 % of consumers or more I’d the premium they’d say, “the premium rules,” and that rules the day…”* (P18, CAC)

A consumer participant further explained that monthly premiums may be more important than the deductible in decision making due to immediate affordability concerns:

*“I think like anybody one of the things you have to think about is what’s affordable, what can I afford monthly premium wise, probably even more so than the…deductible.”* (P38, Consumer)

Many stakeholders thought, though, that other insurance features beyond premiums should also play into health insurance decision making. Not all consumers were aware of how multiple costs and features differed across health insurance plans, which stakeholders felt could set consumers up for high bills if they do need care during the year:

*“[Client’s priorities should be] … all the pricing of course which would be not only premiums but also deductible and the max out of pocket which not all consumers are immediately aware of.”* (P29, CAC)

*“You know um a lot of people don’t even know that it’s, their deductibles and so, you know, insurance uh premiums may be low but the deductible might be high.”* (P9, CAC)

#### Importance of network coverage differs among consumers

Stakeholders recommended excluding some topics that were not the most pertinent from decision support tools. For example, some consumers without current providers might place less importance on hospital or provider networks. As one participant noted:

*“Um, or they’re just like, “oh I haven’t been [to the doctor] in a lot of years,” I don’t care if I have to change. Like most people are pretty indifferent about that [network list], unless they, like, actually have…health issues that they’re concerned about.”* (P6, CAC)

However, others felt network coverage was central to the decision:

*“Uh, I would be very upset if I couldn’t keep my doctor. He’s a great guy. Sometimes when we don’t have money he just brings us in and sees us anyway*.” (P32, Consumer)

*“And then like I said, then I would be looking at, “can I go to the providers that I want to go to,” um, because, I want to go to certain providers, I don’t want to go to other places.”* (P10, CAC)

Stakeholders shared that network coverage information needs may differ for consumers. But, they agreed that providing specific network information relevant to the consumer may help with decision making. For example, stakeholders thought that network coverage might be particularly salient for individuals with existing health conditions:

*“Yeah, wherever you want to be able to go, um are there facilities or physicians in your area, which um here in St. Louis it’s not too much of an issue but it can be a challenge again with people with complicated health concerns that those will fit into the network and the plan.”* (P19, CAC)

### Ways to modify strategies

#### Addressing challenges through plain language principles

Stakeholders recommended utilizing plain language principles to ameliorate challenges with health insurance terminology:

*“Ok, I think the language needs to be simplified. It’s very, very complicated to most people…even me… I’m like “What are they talking about here?”* (P5, Policy)

Stakeholders also supported chunking information into manageable pieces to make the process less overwhelming:

*“I think this is a really traditional model, that’s what you get when you go to, look in the insurance company and everybody disregards that because it’s just too complex and so breaking them down in a smaller format makes sense.”* (P3, Policy)

*“…having it broken down into smaller charts would probably make it less intimidating for people to read.”* (P6, CAC)

*“… And just visually… I think it’s just easier for people, [to] get one subject, um, at a time… I kind of, vote for that.”* (P17, Provider)

“*Um, I would want to see the basics about each plan…otherwise it would just be way too overwhelming and hard to keep all of the information straight.*” (P4, Policy)

Furthermore, engaging graphics might facilitate clear communication about health insurance:

*“Hmm, (laughs), I think more visual aids would be helpful. Um, so something that’s not just words on the screen…”* (P4, Policy)

When looking at one strategy, a policy participant stated:

*“I like the picture, I like the white space. Um, very simple, small bit of information on the first page.”* (P37, Policy)

#### Including comprehensive, tailored narratives to support insurance choices

Many stakeholders felt that narratives or personal stories about how others make health decisions were useful as tools for helping people understand the process of health insurance decision making. In response to viewing our previously developed narratives [4, 9], one consumer said:

*“I think that looking at these, um, the way that these vignettes walk you through somebody’s decision process could be very helpful.”* (P33, Consumer)

*“Yeah I like, I like this [narrative], this is exactly what I was saying I’d like to see somebody, have an example, somebody that’s got a similar situation and find out what they did. Um, if that’s the one they chose well I can look at that and say “yes, that makes sense, you know, that’s what I probably ought to do as well.”* (P34, Consumer)

However, some were concerned that written narratives might take too much time to read, overwhelm consumers with information, or be inaccessible for those with limited literacy.

*“… I think a person like that probably isn’t going to gravitate toward a big block of text and read it. Um…although I think the examples themselves are useful… I just don’t know if I see people actually reading them… especially when they already are taking in so information and they are pressed for time…”* (P29, CAC)

Stakeholders also felt it could be difficult to create narratives to match each individual’s’ unique situation and context:

*“A lot of people have complex enough family situations that there may or may not be just one of the vignettes that matches perfectly with theirs and so then they have to put them all side by side and make those, make those decisions and I think that’s hard.”* (P3, Policy)

*“…[maybe] include someone who is disabled…include someone who is going to be graduating off their parents insurance at 26 or 27…include someone who, because of their citizenship status does not qualify to get Medicaid then they will be under their parents, um, someone who has a kid who lives with their family here but works in a different state…maybe possibly even someone who has a kid in college in a different state but their permanent residence is with their parents, what kind of plan would you buy for that?*” (P27, CAC)

*“[what about] same sex couples....and then the invincible young man kind of thing…and a multi-generational family, you know, where there’s’ like a, mom, or a grandma…we see so many of those types of families…there are those families out there.”* (P10, CAC)

Others suggested creating a mechanism to tailor narratives so as only to present the most relevant ones to consumers:

*“Now so one of my reactions is, my only, so if I had all of these put in front of me, I would actually want somebody to sort of hand me the one (laughs) that fits my situation closest. Um, and so I don’t know if that’s, once again, if you’re sitting with an assister or someone that’s helping you with this, instead of putting all five of these in front of me, if I know I little bit about you as the client, maybe I’m only putting one or two of these in front of you that best [fit] sort of your family situation.”* (P37, Policy)

## Discussion

Our study explored stakeholders’ perceptions of the challenges associated with health insurance enrollment in the ACA Marketplace and gathered their thoughts on information essential to include in health insurance decision support. Unclear terminology, high costs, limited financial literacy of the health insurance decision-makers were cited as common enrollment barriers for consumers, as supported by existing literature [5, 7, 22–27]. Our study advances research in this area by describing key stakeholders’ experiences and recommendations to support individual’s decisions using the ACA Marketplace. These include: acknowledging the fears associated with enrollment commitments, implementing tools to define and clarify important insurance terms, tailoring information to patient characteristics, and facilitating comparisons of various cost information across plans to help consumer decision making.

Stakeholders reported that consumers often express anxiety about the process of making a health insurance decision for the upcoming year. Tradeoffs between cost and coverage are cognitively difficult for consumers to weigh. In addition, consumers are expected to forecast which health insurance features they may use in a 12-month period. Many stakeholders indicated that this process was quite stressful for those new to the insurance market. Supporting consumers’ cognitive and emotional experiences by helping them anticipate potential needs and preferences during the upcoming year and reassuring them about the ability to change in 12 months may be warranted when developing decision support tools.

Health insurance decision support should also help consumers consider costs for the entire year, rather than solely focusing on monthly premiums. Consumers strongly attend to the premium, which may be driving decision making due to immediate affordability limitations. However, consumers may be affected by other health insurance costs throughout the year [28]. Stakeholders cautioned that consumers paying low monthly premiums just to have insurance might not understand that such plans often include high out of pocket expenses, and as a result may find themselves unable to afford care. Increasing awareness of the multiple costs associated with health insurance plans can help prepare consumers for the financial demands they may face throughout the calendar year. Additionally, affordability is a concern and training CACs on how to discuss affordability and subsidy eligibility is needed.

Stakeholders differed in their perceptions of the importance of network coverage to consumers in making health insurance decisions. Some stakeholders felt that network coverage was less important than the overall costs of features such as premiums, copayments, deductibles, and out of pocket maximums, since many low-income consumers will focus on paying bills rather than which providers or hospitals they can use in a plan’s network. However, others advocated for including network coverage information as a priority area. They felt that many consumers could have existing relationship with health care providers whom they trust. In addition, they felt that individuals with chronic conditions may be particularly interested in network coverage due to concerns over disease management. If the only specialty care center in a region is excluded from a plan, for example, an individual could be stuck with high out of pocket bills. Furthermore, use of audio or visual narratives about those in similar situations making insurance decisions may be helpful, as long as they are inclusive enough for a diverse population and/or tailored to individual needs, which may be exceedingly challenging.

As found in previous work [4, 9], stakeholders supported using plain language principles to communicate about health insurance and simplify the enrollment process for consumers. Chunking information, presenting it in simplified, step-by-step formats, and providing concrete examples of how to understand the health insurance terminology could make enrollment less overwhelming and more manageable [24, 29–31]. Relevant graphics could also help by breaking up text with examples [7, 32].

Although many stakeholders felt the narratives presented in our strategies were realistic and relatable, some expressed concerns that written narratives might take too much time, overwhelm consumers with information, or be inaccessible for those with limited literacy. Stakeholders also felt it could be difficult to create narratives to match each individual’s unique situation and context. Health insurance decision support could include narratives that are tailored more closely to individual consumer needs, either through an automated process or by re-formatting them to make it easier for consumers to self-tailor. Audio narratives could also reduce the cognitive burden associated with reading text-based stories.

These findings should be interpreted in the context of study strengths and limitations. Although CAC stakeholders were overrepresented in our sample because of their strong interest in participating in the study and experience with ACA outreach and education, their participation and extensive knowledge of the enrollment process augmented our understanding of study questions. Health care providers may have been underrepresented in our sample because of time commitments and scheduling demands. In addition, they may not be as familiar with the enrollment process because their primary role involves providing clinical care. The qualitative nature of our investigation provided rich, meaningful data about study themes and questions; however, results might not be generalizable beyond the study sample. Future work could investigate interventions to support health insurance decision making using our recommendations.

## Conclusions

This study describes stakeholders’ perceptions of the health insurance enrollment process. Strategies that clarify important insurance terms, provide simple comparisons of costs across plans, and chunk and tailor information could benefit consumers. Addressing fears associated with making the wrong choice may be needed. Considering these challenges and potential solutions when supporting ACA Marketplace enrollment may help consumers to select a health insurance plan that fits their needs.

## Appendix

### Draft Qualitative Interview Guide With Stakeholders

Thanks so much for coming. I am [xxxx]. As you know, we are here as part of a research study to help understand the kind of information people find useful when they make health insurance decisions.

In an earlier project, we asked uninsured individuals what they understood about health insurance terms and details, and what was confusing to them. We developed some tools for communicating about health insurance based on what they told us. We tested these and found that they all worked well for many people, but there were still some challenges many people faced.

We would now like to ask you some questions about the tools we created to help support people’s health insurance decisions so we can make them better. We really appreciate your help, as it is important to our research team to hear from people who know the most about this issue. We’re here to learn from you, so anything you have to share is welcome.

### Background Questions

- Before we begin, please tell me about your role here at [local institution, for institutional stakeholders].
- How well do you think that health insurance decisions are made here with clients?
- Does your organization help clients through the process of making health insurance decisions?

[OR, for uninsured participants]:

- Before we begin, please tell me about your past experiences with health insurance [for uninsured participants].
- Are there key issues that are particularly confusing to you about health insurance?

### Approaching the decision

2. As you know, making a decision about which health insurance plan to choose is quite difficult. How would you go about making this decision to choose which insurance plan is best for you or your family? What process would you go through?

Probes:

- If you were faced with this kind of decision, what would be your first step? Where would you start?
- What about support from someone else? Are there people you would ask for help?

3. What kinds of information would you want if you were considering purchasing health insurance?

### Improving the decision

4. How could the process of making health insurance decisions be improved?

5. What do you think is the biggest challenge people face when making health insurance decisions?

### How much detail is wanted

6. We’d like to know how much detail people want when they get information about health insurance. Some people want lots of details. Some don’t want to be overwhelmed; they might prefer an easy-to-understand chart or hearing from someone who faced a similar decision. What are your thoughts about how much information you would want about health insurance if you were making a decision? (for institutional stakeholders): How much information do your clients want when making a decision about health insurance?

Probes:

- What topics would you or your clients want details about?
- Who would you want to get these information details from? [Probes: If people are silent, ask about health care providers, websites, friends, family, magazines, TV, Google, blogs, Facebook, and so on.] Help us make a list of the top places people would want to get details.
- If we put information on a website, what information should be on the *first page* for everyone to see? What details should be available for people to drill down to?

### Specific suggestions about the tool(s)

7. As I mentioned before, in an earlier project, we developed some tools for communicating about health insurance information to people. As you look at these materials, I’m going to ask you as much as possible to try to *think out loud*: to say what you’re looking at, what you’re trying to do, and what you’re thinking. This will be a big help to us. Sometimes people forget, so I may remind you occasionally.

Also, please don’t worry that you’re going to hurt our feelings. I was not involved in the development of the tools and we are doing this to improve the tools, so we want to hear your honest reactions.

If you have any questions as we go along, just ask them. I may not be able to answer them right away, since we’re interested in what people are thinking about these tools when they don’t have someone sitting next to them to help. But if you still have questions when we’re done, I’ll answer them then.

Here is the first tool we created [will show plain language tool]. As a reminder, it was created for individuals who need health insurance and want to learn more about their options before deciding which health insurance plan to select.

What is your overall reaction to this tool?

What do you like about this tool?

How can it be improved?

[do the same with the visuals and narratives]

9. We want to make these tools available electronically or on the Internet.

[for institutional stakeholders] How would your institution feel about implementing an electronic or Internet based tool that can help support people’s health insurance decisions? What issues should we consider about using electronic tools? What is your institution’s experience using electronic tools to deliver health information to clients?

[for uninsured participants] How would you feel about an electronic or Internet-based tool that supports people’s health insurance decisions? Would that be useful for you? What issues should we consider about electronic tools? What are your experiences using electronic tools to access health information?

### Wrap-up

We’ve covered quite a bit. But, what have we missed that would be important to have available for people as they make a decision about health insurance?

## Abbreviations

ACA: Affordable care act
CAC: Certified application counselor
P: Participant
